# Host Immune Response to Influenza A Virus Infection

**DOI:** 10.3389/fimmu.2018.00320

**Published:** 2018-03-05

**Authors:** Xiaoyong Chen, Shasha Liu, Mohsan Ullah Goraya, Mohamed Maarouf, Shile Huang, Ji-Long Chen

**Affiliations:** ^1^Key Laboratory of Fujian-Taiwan Animal Pathogen Biology, College of Animal Sciences, Fujian Agriculture and Forestry University, Fuzhou, China; ^2^CAS Key Laboratory of Pathogenic Microbiology and Immunology, Institute of Microbiology, Chinese Academy of Sciences (CAS), Beijing, China; ^3^University of Chinese Academy of Sciences, Beijing, China; ^4^Department of Biochemistry and Molecular Biology, Louisiana State University Health Sciences Center, Shreveport, LA, United States

**Keywords:** influenza A virus, immune response, innate immunity, adaptive immunity, immune evasion

## Abstract

Influenza A viruses (IAVs) are contagious pathogens responsible for severe respiratory infection in humans and animals worldwide. Upon detection of IAV infection, host immune system aims to defend against and clear the viral infection. Innate immune system is comprised of physical barriers (mucus and collectins), various phagocytic cells, group of cytokines, interferons (IFNs), and IFN-stimulated genes, which provide first line of defense against IAV infection. The adaptive immunity is mediated by B cells and T cells, characterized with antigen-specific memory cells, capturing and neutralizing the pathogen. The humoral immune response functions through hemagglutinin-specific circulating antibodies to neutralize IAV. In addition, antibodies can bind to the surface of infected cells and induce antibody-dependent cell-mediated cytotoxicity or complement activation. Although there are neutralizing antibodies against the virus, cellular immunity also plays a crucial role in the fight against IAVs. On the other hand, IAVs have developed multiple strategies to escape from host immune surveillance for successful replication. In this review, we discuss how immune system, especially innate immune system and critical molecules are involved in the antiviral defense against IAVs. In addition, we highlight how IAVs antagonize different immune responses to achieve a successful infection.

## Introduction

Influenza viruses belong to the *Orthomyxoviridae* family, which is characterized by a segmented, negative sense, and single-stranded RNA (ssRNA) genome (1). They are categorized into four genera (type A, B, C, and D), among which influenza A virus (IAV) can infect a wide spectrum of animal species (2, 3). The IAV genome is about 13,500 bases long and composed of eight ribonucleoprotein (RNP) units that encode at least 17 distinct proteins, including recently identified NS3, M42, PA-N182, and PA-N155 (4–6). IAVs can be further classified on the basis of the molecular structure and genetic characteristics of hemagglutinin (HA) and neuraminidase (NA) proteins. To date, 16 HA subtypes and 9 NA subtypes have been identified to be circulating in animals and humans (7). Besides, two HA- and NA-like subtypes designating IAV-like viruses (H17N10 and H18N11) have recently been discovered in bats (8). Co-infection with multiple virus strains in individuals can result in re-assortment (antigenic shift) of genes to produce novel subtypes that could give rise to a global influenza outbreak (9). There are approximately 5 million clinical infection cases caused by influenza viruses every year and 250,000–500,000 deaths resulted from the annual epidemics around the globe, particularly in people over 65 years old who account for 90% of all influenza-associated deaths in the USA (10). Moreover, in March 2013, residents in China exhibiting signs of respiratory infection were first reported to be infected by a novel re-assorted IAV of avian-origin, which was isolated from infected patients and identified as H7N9 (11).

There are several important IAV-encoded proteins that have been reported to be associated with the virus pathogenesis and host immune response to the viral infection. It has been revealed that changes at amino acid level in the viral proteins are related to increased disease severity and immune evasion in humans or avian caused by IAVs. For example, HA is the most abundant surface glycoprotein of the virus that has the ability to attach the host cell, causing cellular fusion and viral entry (12). In addition, HA contains epitopes which are key to trigger the production of neutralizing antibodies by B cells. Thus, the epitopes of HA are the dominant determinants that affect viral mutation and recombination mechanisms (13). The high variability of HA allows IAVs to escape from host immune surveillance and results in influenza seasonal epidemics. NA, the second most abundant glycoprotein, cleaves sialic acid (SA) moieties, promotes the release of nascent virions, and facilitates IAV dispersion (14). NA also plays crucial roles in the viral infection and HA-mediated membrane fusion by binding to SA receptors (15, 16). Respiratory epithelial cells constitutively expressed mucin glycoproteins at their surface that include MUC5AC, MUC5B, and MUC1, which play an important role in restricting IAV infection (17–19). For example, these mucin glycoproteins are rich in SA, which act as viral receptor decoys and prevent the viral binding to the target cells (19–21). However, NA can degrade these mucins and thus attenuate their action (21, 22). Moreover, it is shown that amino acid residues 147 and 151 in NA protein are critical for its interaction with SA. For example, D151G mutation in NA is responsible for its binding to avian α2-3 and human α2-6 SA, promoting the association of H3N2 virus with SA receptors. However, this mutation decreases the enzymatic activity of NA needed to detach the HA from its receptors (22, 23).

Nonstructural protein-1 (NS1), a multiple function protein, contains two functional domains (N-terminal RNA-binding domain and C-terminal effector domain). NS1 is a major inhibitor of host innate immune response. For example, it suppresses the production and signaling of type I interferons (IFNs) (24). In addition, NS1 can trigger the apoptosis of human airway epithelial cells *via* a caspase-dependent mechanism during the IAV infection (25). Matrix protein 2 (M2) forms tetrameric proton channels that are responsible to maintain pH across the viral envelope following the viral endocytosis, and help to release the uncoated viral RNP into the cytoplasm and nuclear import to start viral replication. M2 helps to hold the optimum high pH of the trans-Golgi network for HA-induced fusion and prevents premature conformational changes of HA (26). PB1-F2 is encoded by the alternate open reading frame of PB1 gene of IAV. PB1-F2 with N66S mutation binds with mitochondrial antiviral signaling protein (MAVS) and inhibits the initiation of IFNs. The 1918 deadly influenza strain H1N1 and H5N1 with N66S mutation increase the production of pro-inflammatory cytokines and enhance viral replication in the lung (27, 28).

During infection, host innate immunity provides the first line of defense and triggers pro-inflammatory responses (29). Adaptive immunity also plays a critical role in the clearance of viral pathogens during the later stages of infection. Additionally, respiratory mucosal immunity is induced in the related mucosal tissues during the IAV infection and involved in antiviral defense. In spite of several immune mechanisms to neutralize invasive pathogens or restrict viral replication, IAVs still have evolved diverse strategies to evade host immunity and can establish successful infection. Here, we review how host immune system responds to IAV infection and how IAVs evade the host immune surveillance.

## Innate Immune Response to the IAV Infection

### IAVs Target and Enter Host Cells

Influenza A viruses primarily target and infect airway and alveolar epithelial cells, which contain the SA glycans as receptors, thus causing alveolar epithelial injury and eventually failure of gas exchange (30, 31). Hence, human IAV infection may lead to acute respiratory distress syndrome (ARDS) and even death (32, 33). Various subtypes of IAVs have different abilities to attach human upper respiratory tract (URT). For example, H1N1 adsorbs abundant ciliated epithelial cells and goblet cells, whereas H5N1 hardly attaches to these cells in human URT (30). In contrast, H5N1 infects alveolar macrophages as well as alveolar epithelial cells (34, 35). Additionally, human and avian IAVs could target and infect various cells in the lower respiratory tract (LRT). It has been observed that H1N1 and H3N2 attach more abundantly to human trachea and bronchi and adsorb more cell types than H5N1 (36). Of note, low pathogenic (LP) avian IAVs generally do not cause a severe pneumonia because they bind human submucosal gland cells and their mucus which can restrain and remove these viruses before approaching LRT (37). However, high pathogenic (HP) H5N1 is able to infect type II pneumocytes as these cells possess an active metabolism, therefore providing a possibility to develop severe pneumonia (35). Moreover, it has been shown that H5N1 reduces proliferation of infected endothelial cells and causes excessive production of cytokines, leading to the lung damage (38, 39).

Hemagglutinin protein on the viral envelope can recognize SA receptors on the surface of the host cells, which is the most crucial step in the process of IAV invasion into an organism (40, 41). Influenza viruses have two common cellular receptors: SA α-2,3 galactose (SAα-2,3-Gal) and SA α-2,6 galactose (SAα-2,6-Gal) (42). It has been found that human influenza strains preferentially attach to the SAα-2,6-Gal receptor (43). The 2,6 SA receptors are present on respiratory epithelial cells of the human URT, while 2,3 SA receptors are found on epithelial cells of the birds, pigs, and in the LRT of humans (44). Thus, variation of cell surface receptors contributes as a major barrier to cross-species and zoonotic transmissions of influenza virus. Hydrolysis of HA0 precursor gives rise to a dimer HA1–HA2 linked by disulfide bonds. HA1 binds to cellular receptors and HA2 facilitates the fusion of the virus to cellular membrane (45). It is also found that HA of influenza virus binds to C-type lectin as an alternative of SA (46). Virus attachment to host cell induces endocytosis using the clatherin and clavoline-dependent mechanism. Following the endocytosis, release of viral particle is pH-dependent physiological event that occurs at late lysosome (47). Low pH of endosome opens the M2 proton channel for proton flux into the virus and triggers the uncoating and subsequent release of viral RNPs (48). The free viral RNA is imported to the nucleus by interacting with the cellular importin-α/β for replication (49, 50). Although it is recognized that both virus preference for SA receptor and host restriction factors are critical for IAV binding and entry into host cells, further studies are needed to unravel the complicated mechanisms underlying the interaction of IAVs with human airway cells.

### Activation of Innate Immune Signaling upon Intracellular Detection of IAV Infection

The innate immune response is the first line of defense against viral infection which is rapid in response, but nonspecific. During the IAV infection, viral conserved components called pathogen associated molecular patterns (PAMPs) are recognized by host pathogen recognition receptors (PRRs), such as retinoic acid-inducible gene-I protein (RIG-I) and toll-like receptor (TLR), leading to activation of innate immune signaling that finally induces the production of various cytokines and antiviral molecules (51, 52). These PAMPs have certain characteristic of viral RNA that are not shared by cellular RNAs, such as regions of double-stranded RNA (dsRNA) or the presence of a 5’-triphosphate group (53, 54).

**Figure 1:**
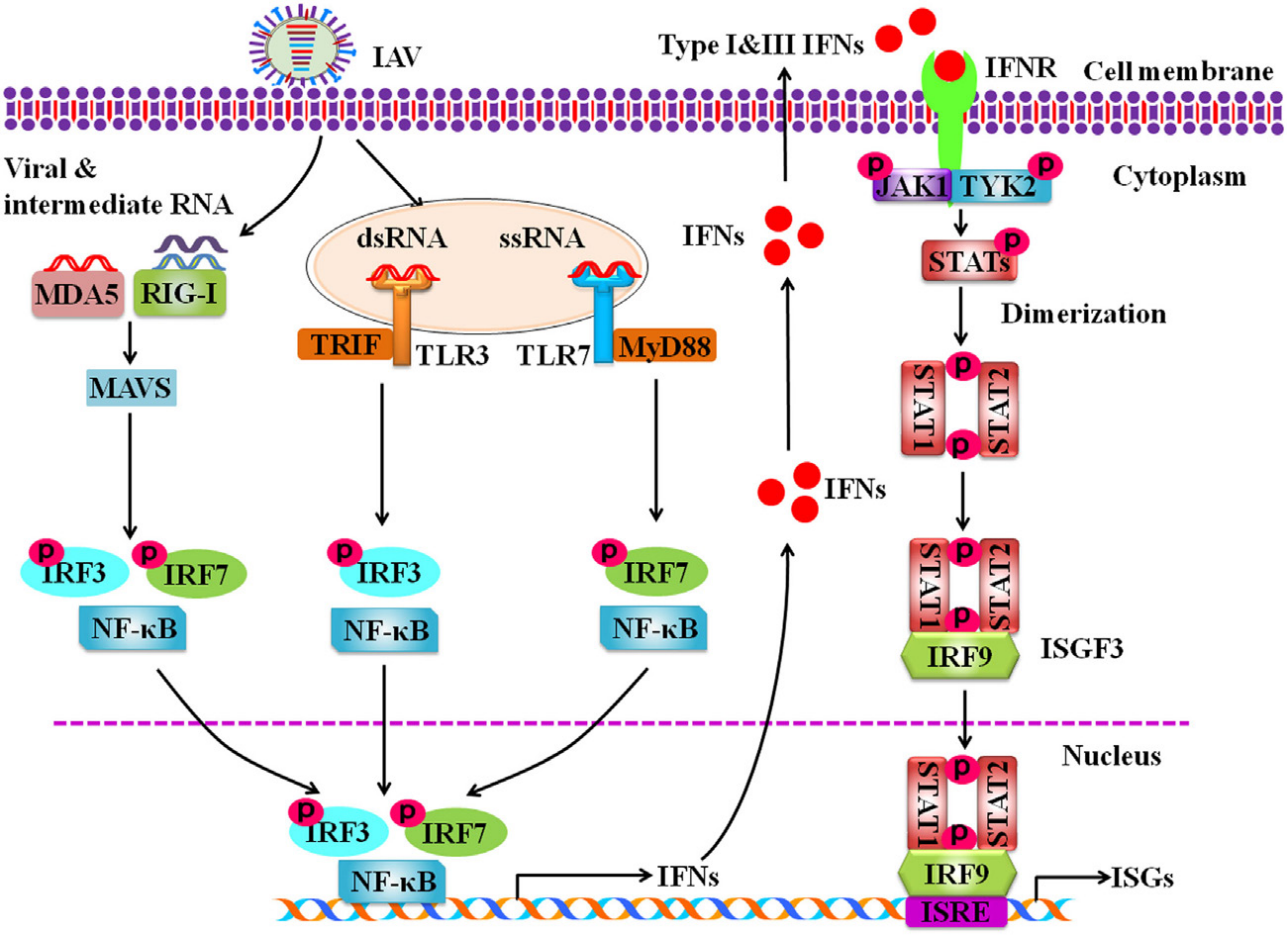
Schematic diagram for innate immune response against influenza A virus (IAV) infection. Intracellular detection of IAV infection by the host pathogen recognition receptors (PRRs) activates transcription factors nuclear factor kappa-light-chain-enhancer of activated B cells, interferon regulatory factor 3 (IRF3), and IRF7. The PRRs include retinoic acid-inducible gene-I protein, melanoma differentiation-associated gene 5, and toll-like receptors. Then these activated transcription factors trigger the expression of type I and type III interferons (IFNs). IFNs secreted by the infected cells interact with their receptors, which results in activation of Janus kinase-signal transducer and activator of transcription signaling pathway that governs the expression of various IFN-stimulated genes.

Pathogen recognition receptors have the ability to distinguish self from non-self molecules within the infected cells. RIG-I is the main receptor to recognize the intracellular ssRNA and transcriptional intermediates of IAVs in the infected host cells (Figure 1). Non-self RNA and transcriptional products of IAVs in the cytoplasm are also sensed by melanoma differentiation-associated gene 5 (55). Following the recognition of PAMPs, RIG-I is activated and its caspase activation and recruitment domains (CARDs) are exposed. Then the CARD is modulated by dephosphorylation or ubiquitination by E3 ligases, such as TRIM-containing protein 25 (TRIM25) (56). Thus, CARD-dependent association of RIG-I and MAVS trigger the downstream transduction signaling at the outer mitochondrial membrane (57). Subsequently, the transcription factors, including interferon regulatory factor 3 (IRF3) and IRF7, and nuclear factor kappa-light-chain-enhancer of activated B cells (NF-κB) are activated, causing the expression of a variety of IFNs and cytokines (Figure 1) (58).

Toll-like receptors are critical PRRs that sense the pathogen outside of cell membrane and internally at endosomes and lysosomes (59). TLRs expressed on cell membrane are TLR1, 2, 4, 5, and 6 that recognize PAMPs derived from bacteria, fungi, and protozoa, while TLR3, 7, 8, and 9 are expressed on the surface of endosomes and lysosomes and exclusively recognize nucleic acid PAMPs derived from various viruses, including IAVs (60–62). TLR3, TLR7, and TLR8 are involved in sensing the IAV components in cytoplasmic endosomes during the virus replication. It is known that TLR3 recognizes dsRNA in endosomes (63). Interestingly, it was shown that TLR3 may recognize recently unidentified RNA structures that are present in phagocytosed cells infected with IAVs (64). In plasmacytoid dendritic cells (pDCs), TLR7 recognizes the ssRNA of the influenza virions that are taken up into the endosomes (65). Then downstream signaling of TLR7 is activated *via* the adaptor protein myeloid differentiation factor 88 in pDCs, which results in the activation of either NF-κB or IRF7 to induce the expression of pro-inflammatory cytokines and type I IFNs, respectively (65). In macrophages and DCs, TLR3 interacts with TIR-domain-containing adapter-inducing interferon-β. Such interaction results in activation of the serine–threonine kinases IκKε (IKKε) and TBK1 that phosphorylate IRF3 to regulate the expression of IFN-β (66). In human monocytes and macrophages, TLR8 is stimulated by its ligand ssRNA, leading to the production of IL-12. However, the relationship between TLR8 and IAV infection has not been defined (67).

Moreover, some NOD-like receptors, such as NOD-like receptor family pyrin domain containing 3 (NLRP3, also known as cryopyrin) and NLR apoptosis inhibitory protein 5, have been observed to be activated upon cellular infection with IAV (68). NLRP3 is expressed by number of cells, including DCs, macrophages, neutrophils, monocytes, and human pulmonary epithelial cells (69, 70). Three signals are required for activation of inflamosome to trigger cytokine production. First, NLRP3 is activated through pathogen detection, which induces the expression of the genes encoding pro-IL-1β, pro-IL-18, and pro-caspase-1 (71). Second, the IAV-encoded M2 ion channel is required to trigger NLRP3 inflammasome activation and cleavage of pro-IL-1β and pro-IL-18 (72). Recently, it was revealed that accumulation of IAV PB1-F2 in the lysosome of macrophages acted as the third signal leading to activation of the NLRP3 inflammasome (73).

### Antiviral Molecules Involved in Innate Immunity against IAV Infection

Activation of specific transcription factors including, NF-κB, IRF3, and IRF7 during IAV infection results in translocation of these factors into the nucleus where they initiate the transcription of genes encoding IFNs and pro-inflammatory cytokines (TNF, IL6, IL1β, etc.). It is well known that type I IFNs, such as IFN-α and IFN-β, and type III IFNs also known as interferon lambdas (IFN-λ1, IFN-λ2, IFN-λ3, IFN-λ4) play important roles in antiviral response in both virus-infected and uninfected cells (74). Infection with IAV induces robust expression of type I and type III IFN genes (75). Following the expression, IFN-α and IFN-β interact with IFN-α/β receptors (IFNAR), while IFN-λs interact with IFNL receptors (IFNLR) in an autocrine or paracrine manner, which activate Janus kinase-signal transducer and activator of transcription (JAK-STAT) signaling pathway. Phosphorylated STAT1 and STAT2 bind with IRF9 to form a complex ISG factor 3 (ISGF3). ISGF3 translocates into nucleus and binds with IFN-stimulated response element, which triggers the transcription of numerous IFN-stimulated genes (ISGs) (Figure 1) (76). Previous studies suggest that type I and type III IFNs provide similar defense against IAV infection in wild-type mice (77). It was further shown that IAV infection induced expression of same type of ISGs in epithelial cells of wild type, IFNAR- or IFNLR-deficient mice (78). Only when both IFN-α/β and IFN-λ receptors in mice were knocked out, the animals failed to restrict non-pathogenic influenza virus (77). In spite of similar role of IFNα/β and IFN-λs to a certain extent, some notable differences exist. For example, it has been observed that mice infected with influenza virus showed higher pulmonary inflammation and mortality after treatment with IFNα, while IFN-λ remained protective (79, 80).

These ISGs target different steps of IAV life cycle. For example, viral entry into cells can be restricted by several ISGs, including Mx family, interferon-induced transmembrane protein family (IFITMs), cholesterol 25-hydroxylase (CH25H), and TRIM proteins. Mx family is comprised of MxA and MxB in human, Mx1 and Mx2 in mice. Mx proteins are produced by various cells, such as hepatocytes, DCs, endothelial cells, and immune cells (81). Mx proteins were the first ISGs identified to restrict IAV infection of mice (82). Recently, a study showed that MxA could retain incoming viral genome in the cytoplasm of human cell (83). In addition, nuclear Mx1 in mice impedes the process of early transcription of IAV activated by the polymerase in the nucleus (84). It is thought that the sensitivity of IAVs to MxA depends on their nucleocapsid proteins, and usually avian strains of IAVs are more sensitive to MxA than human strains (85, 86). However, role of MxB in humans or Mx2 in mice during IAV infection is poorly understood (87).

Interferon-induced transmembrane protein families are known as new ISGs that restrict early viral entry by altering the cellular membrane properties like cell adhesion, fluidity, and spontaneous curvatures (88, 89). It has been found that IFITM proteins restrict the replication of IAVs by interfering virus–host cell fusion following viral attachment and endocytosis (90). Another antiviral ISG, CH25H is an integral element of cellular membranes and upregulated by IFN signaling. CH25H enzymatic activity converts cholesterol to soluble 25-hydroxycholesterol (25HC), which is involved in antiviral defense against enveloped viruses, including influenza virus through blocking viral fusion. Recently, it was suggested that high concentration of 25HC causes physical changes of cellular membrane properties to prevent viral fusion (91). Moreover, previous studies showed that IFN-activated STAT1 bound to the promoter proximal region of the Ch25h gene to stimulate the production of 25HC that enhanced innate immune response against IAV (92, 93). In addition, TRIM proteins play multiple roles in antiviral immunity. TRIM25, an E3 ubiquitin ligase, is considered to regulate the re-localization of RIG-I to mitochondrion and signal transduction to MAVS for innate immune response against the viral infection (94). TRIM22 blocks IAV genome encapsidation and degrades nucleoprotein of IAV by polyubiquitination (95). TRIM32 binds with influenza PB1 RNA polymerase, reduces the polymerase activity, and thus restricts the viral replication (96).

There are increasing number of ISGs that regulate viral mRNA expression and protein translation. For example, zinc finger antiviral protein (ZAP), oligoadenylate synthase and ribonuclease L (OAS-RNase L), PKR, and ISG15 are involved in the regulation of IAV mRNA levels and protein synthesis. ZAPs inhibit the expression of IAV PB2 and PA proteins by reducing the viral mRNA expression and blocking its translation (97, 98). OAS-RNase L can destroy viral RNA in the cytosol of host cells and finally halt the protein synthesis process and viral replication. Mice with reduced expression of RNase L are more prone to influenza virus infection (99). PKR is expressed by all kind of cells and upregulated by type I and type III IFNs (100). PKR expressed in inactive form is activated by influenza virus infection. PKR is a known anti-IAV factor that binds to viral dsRNA and suppresses viral protein synthesis. Genetically deficient PKR mice are highly susceptible to influenza virus (101). ISG15 is an ubiquitin-like protein and restrict viral replication by interfering with virus release and translation of viral proteins (102).

In addition, many other ISGs are also involved in innate immunity against IAV infection. These include viperin, tetherin, and so on (103, 104). It has been shown that overexpression of viperin (also known as RSAD2) restricts the release of influenza virus by affecting the formation of lipid rafts specific microdomains that are particular budding sites of the virus (105). Tetherin is another potential host antiviral factor. In 2008, it was reported that tetherin inhibited retrovirus release (106). Tetherin appeared to limit cellular export of viral progenies by internalizing and degrading them exported to the surface of infected cells (107). It was also found that tetherin restrained the influenza virus by tethering and degrading newly budded viral particles (108). Recently, it has been known that tetherin was able to suppress the budding of several laboratory oriented and seasonal influenza strains, but unable to restrict pandemic influenza A/Hamburg/4/2009 and wild-type influenza virus particles (103, 104, 108).

### Cells Involved in Innate Immunity against the IAV Infection

Airway epithelial cells are the first target of IAVs. These cells produce antiviral and chemotactic molecules that initiate immune responses by rapid recruitment of innate effector cells, such as NK cells, monocytes, and neutrophils. All cell types have their own unique mechanisms to interact with virus-infected cells to limit viral replication, and also prime adaptive immune cells for antigen-specific immunity and memory. Tumor necrosis factor alpha (TNF-α) and IL-1 induce endothelial adhesion molecules, which trigger the migration of innate immune cells, such as macrophages, blood borne DCs, and natural killer (NK) cells to the site of infection.

Alveolar macrophages are critical for limiting viral spread. Activated macrophages phagocytose IAV-infected cells and thus limit viral spread and regulate the following adaptive immune response (109). Monocytes derived from bone marrow precursors circulate in bloodstream. During IAV infection, MCP-1 (CCL-2) produced by infected epithelial cells attracts alveolar macrophages and monocytes *via* their CCR2 receptors (110). NK cells are important cytotoxic lymphocytes of innate immune system to eliminate IAV infection. It has been revealed that lysis of IAV-infected cells is mediated by the binding of IAV-HA with the cytotoxicity NKp44 and NKp46 receptors (111). Expression of IAV-HA on the surface of infected cells is recognition signal for NK cells, and thereby NK cells target and lyse the infected cells (112, 113).

Dendritic cells, the specialized antigen-presenting cells, bridge up the innate and adaptive immune responses during the IAV infection. Adaptive immune response begins when naïve and memory T lymphocytes recognize viral antigens presented by DCs. In the naïve steady state, DCs are orchestrated underneath the respiratory tract, including the airway epithelial tissue, lung parenchyma, and the alveolar spaces of the lungs (114, 115), where they constantly monitor for invading pathogens by their dendrites that are extended to airway lumen through the tight junctions of epithelial cells. Upon infection with IAV, the conventional DCs (cDCs) migrate from lungs to lymph nodes through interaction between CCR7 and its ligand CCL19 and CCL21 (116). In the lymph nodes, cDCs present antigens derived from IAV to T lymphocytes (117, 118). The self-infected DCs degrade the viral protein into immune peptides. Immune peptides (epitopes) in the cytosol are exported to the endoplasmatic reticulum, where they bind with major histocompatibility complex (MHC) class I molecule. Following the binding with epitopes, MHC class I is transported to the cell membrane *via* the Golgi complex for recognition by virus-specific CD8^+^ cytotoxic T cells (CTL). However, viral proteins degraded in endosomes/lysosomes are associated with MHC class II molecule. These complexes are presented on the cell membrane for recognition by CD4^+^ T helper (Th) cells. This process may lead to B cell proliferation and maturation to antibody producing plasma cells (119). In addition, DCs can exert cytolytic activity and contribute to the formation of bronchus-associated lymphoid tissue (BALT) during the IAV infection (120).

## Adaptive Immunity Against the IAV Infection

T cells and B cells play key roles in adaptive immunity against the IAV infection. T cells are mainly known as CD4^+^ T and CD8^+^ T cells. CD8^+^ T cells differentiate into cytotoxic T lymphocytes (CTLs), which produce cytokines and effector molecules to restrict viral replication and kill virus-infected cells. Therefore, T cells are crucial for the restriction of viral infection. Upon infection with IAV, naïve CD8^+^ T cells are activated by DCs migrated from lungs to T-cell zone of the draining lymph nodes, leading to T-cell proliferation and differentiation into CTLs (121, 122). Moreover, type I IFNs, IFN-γ, IL-2, and IL-12 also help CD8^+^ T cells to differentiate into CTLs (123, 124). IFN-λs were also shown to enhance the T-cell proliferation during influenza virus vaccination (125). CTLs decrease the expression of CCR7 and upregulate the expression of CXCR3 and CCR4, which enables their migration from lymph nodes to the lungs where they kill IAV-infected cells.

Mechanism by which CTLs function is well understood. Upon targeting the virus-infected cells, CTLs produce cytotoxic granules that contain molecules like perforin and granzymes (e.g., GrA and GrB). Perforin binds target cells to form pores on the cell membrane that promote passive diffusion of granzymes to induce apoptosis. It has also been found that GrA can restrict virus replication *via* cleavage of viral and host cell proteins that are involved in protein synthesis (126, 127). In addition, CTLs have the ability to induce apoptosis by expressing cytokines, such as TNF, FASL, and TRAIL, which recruit death receptors in IAV-infected cells (128). Post-infection virus-specific CTLs and DCs circulate in blood, lymphoid organs, and the site of infection (117, 129). These memory CTL cells are quick in response to secondary IAV infection, and the activation and differentiation process received during first infection affects their proficiency and efficiency during a secondary infection (130). Although neutralizing antibodies protected from second infection with the same serotype of IAV, CTLs are specific for epitopes in conserved IAV proteins, such as NP, M1, and PA. Therefore, the CTL response is heterosubtypic in nature (131).

Studies have shown that IAV-specific CD8^+^ T cells can last for 2 years in murine models (132). The cytotoxicity of the memory CD8^+^ T cells decreases significantly, which is related to their declined target competence and reduced cytolytic molecule expression (131). Autophagy plays a critical role in the establishment of memory CD8^+^ T cells, as Atg7-deficient mice are unable to form CD8^+^ T cell memory against IAV infection (133). Notably, IAV-specific memory CD8^+^ T cells in the nasal epithelia prevent the spread of the virus from the URT to the lung, thus blocking the development of pulmonary disease (134). Besides, lung-resident memory CD8^+^ T cells can defend against heterologous IAV infection, *via* restraining viral replication and facilitating viral elimination (135). Additionally, lung-resident monocytes support to establish lung-resident CD8^+^ T cell during IAV infection (136).

CD4^+^ T cell is another important type of immune cells that is involved in adaptive immunity against the IAV infection. CD4^+^ T cells can also target IAV-infected epithelial cells through MHC class II and induce MHC class II expression in epithelial cells in murine models (137, 138). Multiple co-stimulatory ligands expressed by CD4^+^ T cells contribute to B cell activation and antibody production, among which CD40 ligand (CD40L) is noteworthy (139). CD40L has been shown to enhance immune response against the highly mutated HA protein of IAV (140). Similar to CD8^+^ T cells, CD4^+^ T cells are activated by DCs that migrate from the lung to the draining lymph nodes during the IAV infection (141, 142). CD4^+^ T cells differentiate into Th1 cells in response to IAV infection, according to their stimulators, including antigen, co-stimulatory molecules, and cytokines secreted by DCs, epithelial cells, and inflammatory cells (143, 144). Th1 effector CD4^+^ T cells express antiviral cytokine, such as IFN-γ, TNF, and IL-2 (145), and activate alveolar macrophages (146). The IL-2 and IFN-γ produced by Th1 cells regulate CD8^+^ T-cell differentiation to clear the viral infection (147, 148). CD4^+^ T cells are also able to differentiate into Th2, Th17, regulatory T cells (Treg cells), follicular helper T cells, and sometimes as killer cells (149). Th2 cells bind to virus-derived MHC class II-associated peptides by antigen-presenting cells and produce IL-4 and IL-13 to promote B cell responses predominantly (150). It has been observed that Th17 and Treg cells are involved in regulating cellular immunity against IAV infection (151). Although it is known that CD4^+^ T cells can direct CD8^+^ T cell responses by secreting various cytokines, the precise roles of CD4^+^ T cells to facilitate and regulate CD8^+^ T cell responses to IAV infection remain elusive, because primary CD8^+^ T cell response against IAV infection could be initiated independently of CD4^+^ T cells in mice (152).

B cells are indispensable for priming the defense against infection with heterosubtypic influenza virus strains. In cooperation with memory T cells, naïve B cells reduce morbidity and promote recovery upon heterosubtypic infection (153). At the same time, non-neutralizing antibodies generated by B cells facilitate viral elimination and accelerate memory CD8^+^ T cell expansion after heterosubtypic infection (153). In addition, IAV-specific antibody-dependent cell-mediated cytotoxicity (ADCC) also plays a role in the cross-reaction against diverse HA subtypes (154). Though IgA is important in the protection against IAV infection in the respiratory tract, IgG is the dominant antibody in this process. Additionally, some studies have implied that IgG could inhibit pathogenesis involving influenza, while IgA is more crucial for the inhibition of transmission of IAVs (155).

Till now, the lifespan and response speed of both memory B cells and plasma cells are foremost in the induction of protective antibody response by IAV vaccines. However, in the elderly, the memory B cells are maintained, but the antibody response is not maintained even upon multiple IAV immunizations. This suggests a potential defect with aging in the development of plasma cells (156). A study has shown that autophagy is involved in maintaining memory B cells to counteract IAV infection; Atg7-deficient mice exhibits loss of memory B cells, causing reduced secondary antibody response to IAV infection and displaying severe lung damage (157).

## Respiratory Mucosal Immunity Against the IAV Infection

### Lymphoid Tissues and Immunoglobins in the Respiratory Tract Involved in Immunity against the IAV Infection

The nasal openings and URT are the main entry sites for IAVs and mucosal immune system also acts as the first line to limit the IAV infection apart from innate immunity. Secretory IgA (s-IgA) and IgM are the major neutralizing antibodies present on mucosa to prevent viral entry. Nasal secretions contain IgA which can neutralize HA and NA of IAVs (129, 158). During primary infection with IAVs, all three major immunoglobulin classes (IgG, IgA, and IgM) are present in mucosal secretion to limit the infection, though IgA and IgM are higher in concentration than IgG (159). It is thought that IgM response is dominant during primary infection, whereas during secondary infection IgG response is dominant for immunoglobin secretion (2, 119, 121). In the URT, mucosal response is induced in the nasopharyngeal-associated lymphoid tissues (NALT) (160–162). When antigens are pinocytosed or phagocytosed by macrophages present on the NALT, they interact with local T and B cells, resulting in development of a large number of IgA Ab-forming cell (IgA-AFC) precursors (163, 164). The primed T and B cells migrate from NALT to the lungs *via* general circulation, where they differentiate into specific IgA-AFC to secrete antiviral antibodies. Thus, NALT appears to be initial inductive site for secretion of s-IgA against IAV infection. In the LRT, mucosal immune responses occur in the BALT (165). BALT is the site for AFC development and production of mucosal s-IgA against IAV infection (166).

### Role of s-IgA Antibody in Defense against the IAV Infection

Secretory IgA is the primary isotype detected at the mucosal surface (167), which contributes to mucosal protection through its distinct ability to remove an agent before it traverses the mucosal barrier and infects the cell (168). By covering the viral surface, s-IgA prevents the influenza virions from adhering to the susceptible cells, and thus inhibits their invading host cells and neutralizes the viruses without complement participation. Investigations have been demonstrated that s-IgA plays vital roles both in protection against homologous IAV infection and in cross-protection against URT infection by the viral variants (169). Generally, parenteral administration of IAV vaccine leads to the generation of serum IgG, but not s-IgA, while s-IgA and IgG are both induced by intranasal administration (168, 170). Further, polymeric s-IgA is involved in defending against influenza in humans. Moreover, the quaternary structure of the polymeric s-IgA seems to play a key role in protecting human URT from influenza, and have more neutralizing capacity against IAVs than dimeric s-IgA (171).

## Escape of IAVs from Host Immune Surveillance

To establish a successful infection, IAVs have evolved multiple strategies to circumvent the host immunity. For example, it is well known that IAV infection triggers robust production of IFNs that induce the expression of numerous antiviral molecules or ISGs. Although IFNs have a strong antiviral activity, they cannot fully control IAV infection due to the virus-mediated suppression of IFNs signaling. The mechanisms by which IAVs escape from host antiviral immune responses are discussed here.

### The Antagonism of Major IAV Proteins

Hemagglutinin of IAVs has been shown to facilitate IFNAR ubiquitination and degradation, reducing the levels of IFNAR, and thus suppressing the expression of IFN-stimulated antiviral proteins (172). It has been described that two discrete antigenic sites, H9-A and H9-B, may provide a novel mechanism for H9N2 virus to counteract humoral immunity (173). In addition, a study has shown that the escape of H5N1 from vaccine-mediated immunity is caused by the addition of N-glycosylation sites on the globular head of HA (174). In contrast, antibody response against NA of IAV cannot inhibit viral infection, but restrain its diffusion, thus lowering the severity of influenza. IAVs employ NA protein to block the recognition of HA by natural cytotoxicity receptors, NKp46, and NKp44 receptors and evade the NKp46-mediated elimination, leading to minimized clearance of infected cells by NK cells (175).

Nonstructural protein-1 of IAVs is the most important IFNs antagonist protein, acting on multiple targets and suppressing the host IFN response. Viral RNA invading the host cell causes RIG-I ubiquitination by a RING-finger E3 ubiquitin ligase named as TRIM25, which is essential for RIG-I signaling pathway to trigger host antiviral innate immunity (94, 176). However, NS1 protein can inhibit the TRIM25-mediated RIG-I ubiquitination, thereby blocking RIG-I activation (177). Moreover, NS1 has an inhibitory effect on protein kinase RNA-activated (also known as protein kinase R, PKR), but the effect relies on the induced expression of vault RNAs (a kind of small non-coding RNA with approximately 100 bases). They are initially described as fornix RNP complex components (178). Through NS1 protein, influenza virus induces the expression of vault RNA that inhibits the activation of PKR and the production of IFNs and ultimately promotes the replication of the virus. In a recent reverse genetic investigation, it was found that after interfering with NS1, the phosphorylation level of PKR dramatically increased, which was attenuated by forced expression of vault RNAs (179). These data indicate that IAV has evolved a critical mechanism by which NS1-mediated PKR inhibition is mediated by upregulation of the host factor vault RNAs that inactivates PKR and blocks the production of downstream effector molecules of IFNs.

In addition, studies have shown that through the interaction with IκB kinases (IKK) α and β, two important kinases in NF-κB pathway, NS1 protein can block the phosphorylation of these kinases and eventually destroy the NF-κB complex predominating in nucleus as well as the expression of downstream genes (180, 181). Also, through the JAK-STAT pathway, NS1 protein can block IFN-mediated downstream signaling pathway and weaken the antiviral effect mediated by the downstream effector molecules induced by IFNs. Specifically, NS1 acts mainly by lowering the phosphorylation levels of STAT1, STAT2, and STAT3, preventing STAT2 from entering into the nucleus to bind to the DNA sequence of ISGs promoter region, leading to reduced expression of ISGs (182). Importantly, NS1 is not only involved in host innate immunity, but also affects adaptive immunity *via* modulating the maturation and the capacity of DCs to induce T cell responses (183). Evidence also indicates that influenza virus NS1 can bind to cellular double-stranded DNA (dsDNA), counteract the recruitment of RNA polymerase II (Pol II) to DNA, and finally block the transcription of IFNs and ISGs (184).

### The Antagonism of Other IAV Proteins

Studies have found that PB1-F2 protein has a mitochondrial positioning signal, *via* interacting with MAVS, to counteract RLR-mediated activation of IFN signaling pathway (185). Investigation on the interaction between the virus and host by systematic biology analysis has revealed that PB2 protein, a member of the viral polymerase complex, also plays roles in IFN antagonism (186). Furthermore, PB2 interacts with the MAVS to evade from the host IFN antiviral response, which is similar to the action mode of PB1-F2 protein (187). Recently, viral M2 protein has been found to interfere with the host autophagy (188, 189). These studies have suggested that viral M2 may inhibit the activation of TLR pathway and the generation of IFNs *via* blocking the host autophagy.

## Conclusion

It is well known that host immune response to IAV infection comprises multiple intricate processes that coordinate together to play significant roles in the protection of host. Given the high mutation rate of IAVs, it is necessary to have effective vaccination strategies that can induce robust production of specific antibodies and long-lived T cell response to defend against the viral infection. Since host innate immunity is also critical for anti-IAV infection, further efforts are needed to utilize the current knowledge and technology to enhance the host innate immunity for control of the disease. While our understanding of the IAV-host interaction has increased profoundly, extensive studies are required to better understand the dynamics of host immune system upon detection of the evolved IAVs. Bridging these gaps will pave the way not only for designing better vaccines and effective vaccination strategies but also for developing novel antiviral agents.

## Author Contributions

XC, SL, and MG performed systematic literature review and wrote the manuscript. MM and SH revised the manuscript. J-LC organized and provided the frame for the manuscript and critically revised the manuscript. All authors read and approved the final manuscript.

## Conflict of Interest Statement

The authors declare that the research was conducted in the absence of any commercial or financial relationships that could be construed as a potential conflict of interest. The reviewer HZ and handling editor declared their shared affiliation.
